# Photoprotection by photoinhibitory and PSII-reaction centre quenching controls growth of *Ulva rigida* (Chlorophyta) and is a pre-requisite for green tide formation

**DOI:** 10.1007/s00425-024-04389-z

**Published:** 2024-04-05

**Authors:** Ralf Rautenberger, Catriona L. Hurd

**Affiliations:** 1 Department of Botany, University of Otago, 464 Great King Street, Dunedin, 9016 New Zealand; 2 Division of Food Production and Society, Norwegian Institute of Bioeconomy Research (NIBIO), P.O. Box 115, 1431 Ås, Norway; 3 Institute for Marine and Antarctic Studies (IMAS), University of Tasmania, 20 Castray Esplanade, Battery Point, Hobart, TAS 7001 Australia

**Keywords:** Eutrophication, Macroalgal blooms, Nitrate reductase, Photosynthesis, Photoprotection, *Ulva*

## Abstract

**Main Conclusion:**

The combined photoinhibitory and PSII-reaction centre quenching against light stress is an important mechanism that allows the green macroalga *Ulva rigida* to proliferate and form green tides in coastal ecosystems.

**Abstract:**

Eutrophication of coastal ecosystems often stimulates massive and uncontrolled growth of green macroalgae, causing serious ecological problems. These green tides are frequently exposed to light intensities that can reduce their growth via the production of reactive oxygen species (ROS). To understand the physiological and biochemical mechanisms leading to the formation and maintenance of green tides, the interaction between inorganic nitrogen (N_i_) and light was studied. In a bi-factorial physiological experiment simulating eutrophication under different light levels, the bloom-forming green macroalga *Ulva rigida* was exposed to a combination of ecologically relevant nitrate concentrations (3.8–44.7 µM) and light intensities (50–1100 µmol photons m^−2^ s^−1^) over three days. Although artificial eutrophication (≥ 21.7 µM) stimulated nitrate reductase activity, which regulated both nitrate uptake and vacuolar storage by a feedback mechanism, nitrogen assimilation remained constant. Growth was solely controlled by the light intensity because *U. rigida* was N_i_-replete under oligotrophic conditions (3.8 µM), which requires an effective photoprotective mechanism. Fast declining Fv/Fm and non-photochemical quenching (NPQ) under excess light indicate that the combined photoinhibitory and PSII-reaction centre quenching avoided ROS production effectively. Thus, these mechanisms seem to be key to maintaining high photosynthetic activities and growth rates without producing ROS. Nevertheless, these photoprotective mechanisms allowed *U. rigida* to thrive under the contrasting experimental conditions with high daily growth rates (12–20%). This study helps understand the physiological mechanisms facilitating the formation and persistence of ecologically problematic green tides in coastal areas.

## Introduction

Marine macroalgae or seaweeds are the dominant primary producers and habitat-formers of temperate coastal regions worldwide, supplying ~ 50% of energy to higher trophic levels and providing habitat, food and shelter for invertebrates and fish (Bartsch et al. 2008; Christie et al. 2009; Iken 2012). Since recent estimates showed that macroalgae alone account for 20% of the coastal net primary production worldwide, they have an impact on the global carbon cycling and possibly climate change (Duarte et al. 2022). However, global- and regional-scale climate change factors, such as ocean acidification, ocean warming and eutrophication, are great threats to many seaweed communities, which, therefore, will undergo substantial ecological reorganisations with changes in net primary production (Harley et al. 2012; Duarte et al. 2022). In coastal environments with reduced water exchange (e.g. in lagoons, fjords, estuaries and bays), eutrophication can cause rapid proliferation of opportunistic green macroalgae, such as *Ulva*, *Chaetomorpha* and *Cladophora*, which is an increasing global problem (Bischof et al. 2006; Teichberg et al. 2010; Smetacek and Zingone 2013; Perrot et al. 2014; Nelson et al. 2015; Wallace and Gobler 2015; Hiraoka 2021). Green tides or green macroalgal blooms are densely arranged mats of green macroalgae, which often consist of ten to hundreds of thousands of tonnes of fresh biomass. There are steep gradients of light, oxygen and nutrients within these dense mats, creating different physiological conditions within a few centimetres (Malta et al. 2003; Bischof et al. 2006). Anoxic conditions in the water column due to bacterial decomposing of floating *Ulva* blooms stimulate the microbial production of H_2_S, which accumulates in the sediment. The release of the toxic H_2_S gas from the sediment causes the death of benthic and planktic organisms. This is not only an ecological disaster, but it also leads to economic losses in the affected regions (Smetacek and Zingone 2013).

Bloom-forming green macroalgae have morphological and physiological properties (i.e. high surface area-to-volume ratios and fast nutrient uptake rates) that allow them to exploit the available inorganic nitrogen (N_i_) sources efficiently and to ‘outcompete’ other marine macrophytes and even fast-growing phytoplankton (Rosenberg and Ramus 1984; Runcie et al. 2003; Han and Liu 2014). Because *Ulva* synthesises (almost) exclusively somatic, photosynthetically active tissues, species of this genus can reach high daily growth rates by dividing up to twice a day under optimal abiotic environmental conditions (Hiraoka et al. 2020; Rautenberger 2022).

An efficient nitrogen physiology [i.e. high uptake and assimilation rates at low inorganic nitrogen (N_i_) concentrations] is crucial to the formation of green tides (Naldi and Viaroli 2002; Van Alstyne et al. 2015). After nitrate (NO_3_^–^) enters the cells by specific transport systems, it is either directly assimilated or stored internally in the vacuoles (Runcie et al. 2003; Lartigue and Sherman 2005; Kennison et al. 2011). *Ulva* has a comparatively low storage capacity for nitrate, which reaches only up to 500 µmol g^−1^ DW in *Ulva rigida*, reducing its capacity of overcoming periods of nitrogen deficiency to only a few days (Borum 1996; Naldi and Viaroli 2002). The first step of nitrate incorporation is catalysed by nitrate reductase (NR) in the cytosol (Berges 1997). To reduce nitrate to nitrite (NO_2_^–^), reduction power provides two electrons that derive from the mitochondrial respiratory carbon flow to NR (Huppe and Turpin 1994). In contrast to the processes in higher plants and microalgae in which both NADH and NADPH act as electron donors of NR, NADH is utilised exclusively in *Ulva* (Huppe and Turpin 1994; Hurd et al. 1995). The subsequent reduction of nitrite to ammonium (NH_4_^+^) by nitrite reductase (NiR) in the chloroplast requires six electrons that are provided by the photosynthetic electron transport as reduced ferredoxin (Fd_red_). Another two photosynthetic electrons are needed for the assimilation of ammonium by glutamate synthase (GS) to produce amino acids (Huppe and Turpin 1994). In higher plants, ~ 8% of the photosynthetically transported electrons are used for nitrogen assimilation (Fridlyand and Scheibe 1999). Since these chloroplast-based steps depend on photosynthesis, a modulation of either of these primary physiological processes may thus affect the other.

Green tides of *Ulva* floating on the sea surface or laying as mats on shores are frequently exposed to extreme and rapidly changing radiation regimes during the day. Hence, the light intensities reaching *Ulva*’s surface throughout a day can range from being limited to excessive for photosynthesis, which affects growth (Cruces et al. 2019). At light intensities below or close to their photosynthetic saturation (*E*_k_ = 300–600 µmol photons m^−2^ s^−1^), the majority of light energy absorbed by the light-harvesting complexes of PSII (i.e. LHCII) is utilised by carbon assimilation in the Calvin–Benson cycle to produce carbohydrates for growth (Beer et al. 2000; Rautenberger et al. 2015). Beyond this critical point, however, most of the absorbed light energy cannot be used for carbon assimilation, resulting in an activation of photoprotective mechanisms to avoid photodamage (Wilhelm and Selmar 2011). This usually occurs on sunny days, particularly during midday in summer, when the ground-level light intensities reach more than 1000 µmol photons m^−2^ s^−1^. For example, the mean summer (December–February) light intensities in Dunedin, southeastern New Zealand, ranged between 1346 and 1947 µmol photons m^−2^ s^−1^ in 2012 (R. Rautenberger, unpublished data).

Different photoprotective mechanisms become active under excess light. As an early response to light stress, the majority of the excessively absorbed light energy is dissipated by non-photochemical quenching (NPQ) mechanisms (e.g. the xanthophyll cycle), the activity of photosystem II (PSII) is down-regulated to change the photosynthetic electron transport rate (ETR) (Wilhelm and Selmar 2011). In *Ulva*, the cyclic electron transport around PSII might also become a crucial photoprotective mechanism when the activity of the xanthophyll cycle is limited or non-existent (Franklin and Badger 2001). Under prolonged light stress, the light absorption capacity is reduced by decreasing the sizes of the light-harvesting antennae attached to PSII (Yamazaki et al. 2005; Mou et al. 2013; Zhang et al. 2013). Nevertheless, despite the efficiency of these photoprotective mechanisms, photosynthesis often becomes ‘super-saturated’ under light stress. Photosynthesis generates reactive oxygen species (ROS) in excess by transferring electrons to molecular oxygen (i.e. the Mehler reaction) via Fd_red_ (Asada 1999; Cruces et al. 2019). If ROS is not sufficiently ‘detoxified’ by the network of metabolic antioxidants (e.g. glutathione) and ROS-scavenging enzymes (e.g. superoxide dismutase, SOD) of the Mehler-ascorbate peroxidase pathway, highly reactive peroxides (O_2_^2–^) and free radicals (e.g. ˙OH) oxidise pigments, proteins, lipids and nucleic acids (Bischof and Rautenberger 2012). Although SOD detoxifies superoxide anions, the produced hydrogen peroxide (H_2_O_2_) is removed by ascorbate peroxidase with water as end-product of the reaction (Bischof et al. 2006; Cruces et al. 2019). In addition, *Ulva rigida* excretes H_2_O_2_ produced through photosynthesis under light stress in its surrounding environment (Collén et al. 1995).

The consumption of photosynthetic electrons by certain physiological processes might become an important strategy to protect algae against high light stress when the photosynthetic light and carbon reactions become unbalanced (Wilhelm and Selmar 2011). Amongst them, the stimulation of N_i_ assimilation (e.g. resulting from eutrophication) could require an increased amount of photosynthetic electrons with consequences for the ROS production at PSI. The availability of fewer of these electrons is expected to reduce the production of ROS under high light stress, which could sustain high growth rates of green, bloom-forming macroalgae such as *Ulva*. However, despite mechanistic interactions between N_i_ assimilation and photosynthesis, their impact on growth is not yet well understood in green macroalgae (Cabello-Pasini and Figueroa 2005; Xu et al. 2014). Increased N_i_ assimilation can also affect macroalgal photoacclimation by providing N-rich compounds (e.g. amino acids) for the biosynthesis of proteins during repair processes of damaged D1 proteins when the PSII repair cycle is active (Henley et al. 1991; Bouchard et al. 2006). Therefore, increased N_i_ input by eutrophication may help *Ulva* to respond better to periods of light stress and thereby sustain its fast growth for the formation and maintenance of green tides.

The aim of this study was to gain understanding of the dynamics of green tides by investigating the interaction between nitrogen assimilation and photosynthesis on growth of the bloom-forming green macroalga *U. rigida*. Eutrophication in coastal marine ecosystems was simulated in the laboratory by transferring *U. rigida* from low to higher, ecologically realistic seawater nitrate concentrations under different light intensities. It is hypothesised that the stimulation of nitrate assimilation by eutrophication reduces both photoinhibition of photosynthesis and ROS production under light stress due to higher electron demands. Moreover, higher nitrate assimilation rates may also support photoacclimation by providing N-rich cell compounds for biosynthetic repair processes. Consequently, eutrophication under high light stress has a potential to sustain high growth rates of *U. rigida* as a pre-requisite for its proliferation. The present study helps explain how two of the most prominent environmental factors, light and N_i_, control the formation and maintenance of green tides of *Ulva* in coastal areas. This can be instrumental in the management of harmful green tides in coastal marine ecosystems.

## Materials and methods

### Macroalgal material

A male gametophytic individual of the green macroalga *Ulva rigida* C.Agardh (Chlorophyta; strain SBDN 247, for details see Rautenberger et al. 2015) was collected in October 2011 from the upper subtidal (3 m below sea surface) at Aramoana Spit (Otago Harbour), South Island, New Zealand (45°47ʹS, 170°42ʹE) and transported in a dark box to the laboratory in the Department of Botany, University of Otago. Macroalgal discs of 3 cm^2^ each (~ 25 mg FW) were cut from the thallus and raised as clonal stock cultures in 5 L of continuously aerated seawater in cylindrical glass jars over eight months. Seawater (salinity 34, pH 8.05), collected at Portobello Marine Laboratory (Otago Harbour), was enriched with a third of the full ion strength of the phosphate, iron-EDTA, trace metals II and vitamin stock solution of the ESNW formula and adjusted to pH 7.97 (Berges et al. 2001). Clonal stock cultures were kept at 5 μM of nitrate and 50 μmol photons m^−2^ s^−1^ (Philips TLD 18W/840 Cool White; Philips, Amsterdam, The Netherlands) in a 12h:12h light:dark cycle at 12 ± 1 °C in a climate-controlled cabinet (Contherm Scientific Ltd., Lower Hutt, New Zealand).

### Experimental set-up

The entire experiment, which consisted of all treatment combinations (see below), was repeated three times within four weeks, resulting in three independent replicates (*n* = 3). For each experimental cycle, 144 *Ulva* discs of similar size (3 cm^2^, 25 mg FW) were cut from the clonal stock cultures and evenly distributed amongst nine cylindrical, acid-washed (10% HCl) glass jars, containing 5 L of nutrient-enriched seawater each (see above). The seawater was continuously aerated with filtered air (0.45 µm) from the bottom of the jars and changed each morning at the same time (between 9:30 and 10:30). *Ulva* discs were exposed to 52 ± 1 μmol photons m^−2^ s^−1^ (*n* = 3; 12h:12h light:dark cycle) and incubated at 3.8 ± 0.6 µM nitrate (*n* = 8) at 12 ± 1 °C in a climate-controlled walk-in cabinet (Contherm Scientific Ltd.). Light was provided from the top of the cabinet by ten quartz metal halide lamps (HPI-T Plus 400 W, Philips).

After three days of pre-experimental incubation to acclimate *Ulva* to these growth conditions, the cylindrical jars containing 16 *Ulva* discs each in 5 L of aerated seawater were transferred to one of the nine possible experimental conditions of both factors (light × nitrate) with their three levels (low light, LL: 52 ± 1 µmol photons m^−2^ s^−1^; saturation light, SL: 612 ± 9 µmol photons m^−2^ s^−1^; high light, HL: 1,111 ± 22 µmol photons m^−2^ s^−1^; nitrate: 3.8 ± 0.6 µM, 21.7 ± 1.0 µM, 44.7 ± 4.2 µM; Table 1). Seawater phosphate and ammonium concentrations remained constant throughout the experiment at 7.4 ± 0.9 µM and 0.8 ± 0.2 μM, respectively. The experiment was performed for three days to study the effects of the nitrate assimilation on short-term photoacclimation. The position of the jars was changed randomly in each experimental cycle. The experiment was performed at 12 ± 1 °C. Macroalgal samples for biochemical analyses were taken on the first and third day of the experiment, frozen with liquid nitrogen and stored either at – 20 °C (for pigment analysis) or – 80 °C (for enzyme analyses). Photosynthetic measurements were performed before the experiment started, after eight hours and before the experiment ended on the third day.

**Table 1:** The nine experimental conditions to which *Ulva rigida* was exposed to over three days at 12 ± 1 °C (see text for details)

Experimental condition	Light intensity (µmol photons m^−2^ s^−1^)	Nitrate concentration (µM)
LL × low NO_3_^–^	52 ± 1	3.8 ± 0.6
LL × medium NO_3_^–^	52 ± 1	21.7 ± 1.0
LL × high NO_3_^–^	52 ± 1	44.7 ± 4.2
SL × low NO_3_^–^	612 ± 9	3.8 ± 0.6
SL × medium NO_3_^–^	612 ± 9	21.7 ± 1.0
SL × high NO_3_^–^	612 ± 9	44.7 ± 4.2
HL × low NO_3_^–^	1,111 ± 22	3.8 ± 0.6
HL × medium NO_3_^–^	1,111 ± 22	21.7 ± 1.0
HL × high NO_3_^–^	1,111 ± 22	44.7 ± 4.2

Mean values ± SD (for light intensity *n* = 3; for nitrate concentration *n* = 8–9)

*LL* low light, *SL* saturation light, *HL* high light treatment, *NO*_*3*_^*–*^ nitrate

### Macroalgal growth

Growth was determined by comparing the total FW of *Ulva* discs in each jar at the beginning and the end of the experiment. After the discs were blotted dry, FW was determined using a 3-point balance (Sartorius, Göttingen, Germany). Relative growth rates (RGRs, in % d^−1^) were calculated after Lüning (1990).

### Nitrate uptake rates

Nitrate uptake rates were measured on days 1 and 3 of the experiment. After seawater had been changed in each 5 L glass culture jar in the mornings to avoid nutrient limitation, initial water samples (10 mL at t_0_) were taken before *Ulva* discs were added again. Further water samples (10 mL) were taken 30, 60, 120, 240, 360 and 480 min after the seawater had been renewed. Samples were stored in 10% HCl-washed plastic tubes at – 20 °C until analysis. Nitrate, ammonium and phosphate concentrations in seawater were determined colorimetrically using a ‘QuikChem 8500 series 2’ flow-injection analysis (FIA) system (Lachat Instruments, Loveland, CO, USA) after Parsons et al. (1984). The average nitrate uptake rate (in μmol g^−1^ FW h^−1^) of each replicate over the 480 min sampling period was calculated from plots of nitrate concentration *vs* time using a linear function. The slopes representing the disappearance of nitrate from the seawater by uptake of *Ulva* were divided by the total macroalgal FW in a jar during the sampling period and multiplied with the volume of seawater in a jar at the beginning of sampling (i.e. 5 L).

### Nitrate reductase activity

Frozen algae (– 80 °C) were ground to a fine powder (12–62 mg; Mini-Beadbeater-96, BioSpec Products, Bartlesville, OK, USA) in liquid nitrogen. NR was extracted from crude extracts (1.0 mL 10 mg^−1^ FW) in 200 mM Na_2_HPO_4_/NaH_2_PO_4_ buffer (pH 7.9), containing 3% (*w*/*v*) BSA, 2 mM DTT, 0.3% (w/v) polyvinylpyrrolidone, 5 mM Na_2_–EDTA and 1% (v/v) Triton X-100. Extracts were not centrifuged (Hurd et al. 1995).

For the NR assay, 100 µL of the crude extract was added to 290 µL of 200 mM Na_2_HPO_4_/NaH_2_PO_4_ buffer (pH 7.9), 50 µL of 2 mM NADH (freshly prepared), and 10 µL of 1 mM FAD (freshly prepared). The assay was started by adding 50 µL of 100 mM KNO_3_. After a 15 min incubation at 12 ± 1 °C, the reaction was stopped by precipitation with 250 µL of 1M Zn-acetate at room temperature (RT). For control (intracellular nitrate), 250 µL of 1M Zn-acetate was added to the reaction mixture before the starter was added and identically treated as samples afterwards (Young et al. 2005). After the precipitate was centrifuged (600 *g*, 15 min, RT), 500 µL of 58 mM sulphanilamide (dissolved in 10% HCl) was added to 500 µL of the clear supernatant. Afterwards, 500 µL of 3.9 mM N-(1-naphthyl)ethylenediamine (NED; dissolved in H_2_O) was added to produce a pink azo dye that was measured photometrically at 543 nm. Nitrite concentration (in U g^−1^ FW) was calculated using a standard curve of known nitrite concentrations treated identically with sulphanilamide and NED.

### Photosynthesis

Photosynthetic performance was measured using a PAM-2000 chlorophyll fluorometer (Walz GmbH, Effeltrich, Germany) at 12 ± 1°C (Rautenberger et al. 2009). Minimum (F_0_) and maximum fluorescence (Fm) were measured after 5 min of dark adaptation and maximum PSII-quantum yield, i.e. Fv/Fm, was calculated (Schreiber et al. 1995). Before dark adaption, a 5-s far red light was applied (30 µmol m^−2^ s^−1^). Electron (e^–^) transport rate *vs* irradiance (i.e. ETR-*E*) curves were subsequently recorded by incrementally increasing actinic light intensities after applying a saturation pulse (> 9000 µmol photons m^−2^ s^−1^, 0.8 s) every 30 s. ETRs were calculated by multiplying incident actinic light intensities (*E*, in µmol photons m^−2^ s^−1^), the proportion of *E* that was absorbed by one *Ulva* disc, the fraction of absorbed actinic light that was most probably received by PSII (i.e. 0.5) and the PSII-operating efficiency (Grzymski et al. 1997; Baker 2008). Maximum ETR (ETRmax, in µmol e^–^ m^−2^ s^−1^), the light saturation point of photosynthesis (*E*_k_, in µmol photons m^−2^ s^−1^), and the initial slope of the ETR-*E* curves (α, in µmol e^–^ µmol^−1^ photons) were estimated from ETR-*E* curves fitted to the model of Jassby and Platt (1976) using R version 2.15 (R Core Team, http://www.R-project.org) according. NPQ was calculated from maximum fluorescence yields of dark- (Fm) and light-adapted (Fm′) *Ulva* discs measured at 497 µmol photons m^−2^ s^−1^.

### Pigment analysis

Chlorophylls of frozen samples (– 20 °C) were extracted in 100% N-N-dimethylformamide (DMF; BDH Laboratory Supplies, Farham, Nottingham, UK) in the dark at 4 °C for 24 h. Chl *a* and *b* were analysed spectrophotometrically (Ultrospec 3000, Pharmacia Biotech, Cambridge, UK) at 663.8 and 646.8 nm at RT with 100% DMF as reference. Readings at 750.0 nm were used as a correction factor for scattered light. Both Chl *a* and *b* contents (in μg mg^−1^ FW) were calculated according to Porra et al. (1989).

### Antioxidant enzyme activities

Frozen samples (– 80 °C, 35–62 mg FW) were ground to a fine powder in liquid nitrogen using mortar and pestle. Antioxidant enzymes were extracted with 1 mL of 100 mM KH_2_PO_4_ (pH 7.5/KOH/4 °C) containing 1 mM K_2_-EDTA, 0.1% (v/v) Triton X-100 and Complete Protease Inhibitor Cocktail tablets (EDTA-free, 1 tab 50 mL^−1^; Roche Diagnostics, Mannheim, Germany). After centrifugation (13,000 *g*, 20 min, 4 °C), aliquots of the supernatants were frozen with liquid nitrogen and stored at – 80 °C.

Total SOD activities were determined according to the xanthine oxidase-cytochrome c reduction method of McCord and Fridovich (1969), as described for *Ulva* by Rautenberger and Bischof (2006). Fifty microliters of the crude extract was added to 920 μL of the assay mixture (50 mM KH_2_PO_4_, pH 7.8/KOH/25 °C, 0.1 mM K_2_-EDTA, 0.01 mM cytochrome c and 0.05 mM xanthine). The assay was started by adding 30 μL of xanthine oxidase (Sigma-Aldrich, Munich, Germany) to the assay mixture and the increase in absorbance was recorded photometrically at 550 nm (Ultrospec 3000, Pharmacia Biotech). Xanthine oxidase was adjusted to give an increase in absorbance of 0.025 ± 0.005 min^−1^ at 550 nm and 25 °C in the blank. Total SOD activities (in U mg^−1^ protein) were calculated using an inhibition curve with purified Fe-SOD (Sigma-Aldrich). One unit of SOD activity is defined as the amount of enzyme required to inhibit the rate of cytochrome c reduction by 50% in 3 mL (McCord and Fridovich 1969).

Total glutathione reductase (GR) activities were analysed after Gutterer et al. (1999): 20 μL of 10 mM NADPH (freshly prepared in 100 mM KH_2_PO_4_, pH 7.0/KOH/20 °C) and 100 μL of 10 mM oxidised glutathione (GSSG; dissolved in H_2_O) were added to 840 μL of 100 mM KH_2_PO_4_ (pH 7.0/KOH/20 °C). The reaction was started after adding 40 μL of the crude extract to the assay mixture. The decrease in absorbance was followed photometrically at 340 nm and 20 °C. GR activity (in U mg^−1^ protein) was calculated using the extinction coefficient for NADPH of 6220 M^−1^ cm^−1^.

The total soluble protein content in crude extracts was determined photometrically at 595 nm (Bradford 1976) and calculated by comparing it with a standard curve of BSA (ICPbio Ltd, Auckland, New Zealand).

### Statistical analysis

Statistically significant differences within and between growth light intensities and seawater nitrate concentrations were tested with a model I (fixed effects) two-way ANOVA, one- and two-way repeated measures ANOVA (ANOVA-RM), and a multivariate analysis of variance (MANOVA) using the statistical software JMP Pro versions 10.0 and JMP 11.0 (SAS Institute Inc., Cary, NC, USA). The Shapiro–Wilk *W* test and the Levene test were used to test on normal distribution and homoscedasticity, respectively. Log transformations were performed when data were not normally distributed. Tukey–Kramer Honestly Significantly Different (HSD) test was used as a post hoc test. A 5% significance level (*P* = 0.05) was applied in all tests.

## Results

### Macroalgal growth

**Fig. 1:**
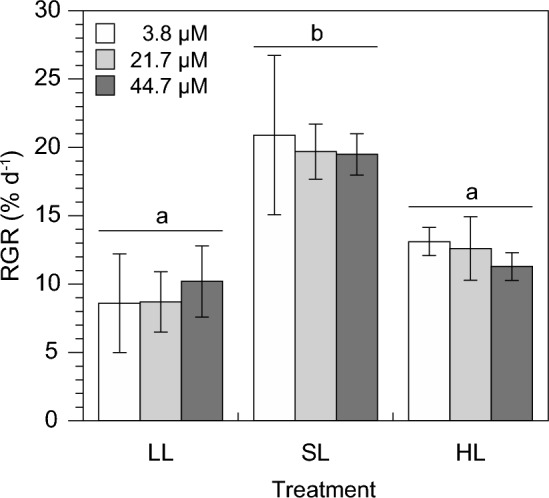
Relative growth rates (RGR) of *Ulva rigida* after three days of exposure to different growth light intensities and nitrate concentrations in seawater. Treatments: LL: 52 µmol photons m^−2^ s^−1^; SL: 612 µmol photons m^−2^ s^−1^; HL: 1,111 µmol photons m^−2^ s^−1^; nitrate concentration: 3.8 µM nitrate (white columns), 21.7 µM nitrate (light grey columns) and 44.7 µM nitrate (dark grey columns). Error bars represent SDs of three replicates per treatment (*n* = 3). Different lower case letters above the columns indicate statistically significant differences of means between treatments (*P* < 0.001, 2-way ANOVA, Tukey HSD)

Growth of *Ulva rigida*, measured by the increase in FW over three days, was strongly controlled by the growth light intensity (*P* = 0.0002, 2-way ANOVA) rather than by nitrate concentration (*P* = 0.8222, 2-way ANOVA) or an interaction between both factors (*P* = 0.8346, 2-way ANOVA; Fig. 1). Relative growth rates (RGRs) of the low light (9.1 ± 0.9% d^−1^) and high light-grown algae (12.3 ± 0.9% d^−1^) were similar but lower than those of *U. rigida* exposed to the saturating light intensity (20.0 ± 0.7% d^−1^).

### Nitrate uptake rates

**Fig. 2:**
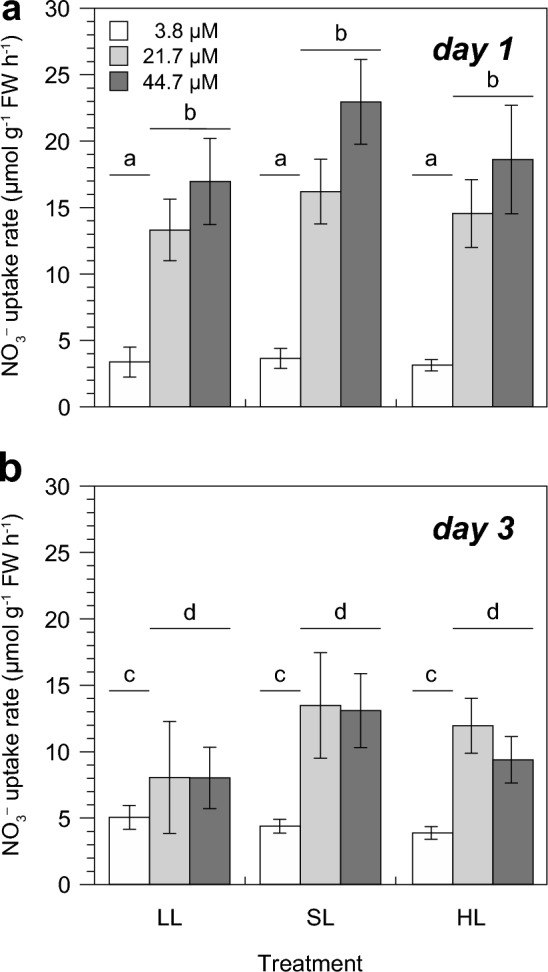
Average nitrate uptake rates of *Ulva rigida* over 480 min at different seawater nitrate concentrations and growth light intensities (**A**) on the first and (**B**) the third day of the experiment. Error bars denote SDs of three replicates per treatment (*n* = 3). Different lower case letters above the columns indicate statistically significant differences between treatments (*P* < 0.05, 2-way ANOVA with repeated measures). For details of experimental treatments see Fig. 1 and Table 1

In all nitrate concentrations and light intensities tested, uptake rates were constant over the 480 min incubation period indicated by the continuous linear decrease in seawater nitrate concentrations (data not shown). Nitrate uptake rates of *U. rigida* changed only in response to the initial nitrate concentration in seawater (i.e. 3.8, 21.7 and 44.7 µM) over the course of the experiment (*P* < 0.0001, ANOVA-RM), but not due to the exposed light intensities (*P* = 0.9034, ANOVA-RM). On the first day, nitrate uptake of *U. rigida* was uniform across all light intensities at the initial concentration of 3.8 µM (3.1–3.6 µmol nitrate g^−1^ FW h^−1^) and increased by 20 to 50% to similar rates ranging between 3.9 and 5.1 µmol nitrate g^−1^ FW h^−1^ two days later (Fig. 2A, B). At initial seawater nitrate concentrations of 21.7 and 44.7 µM, nitrate uptake rates were significantly higher than at 3.8 µM under all light conditions (13.3–23.0 µmol nitrate g^−1^ FW h^−1^) on day 1. They decreased equally to 8.0–13.5 µmol nitrate g^−1^ FW h^−1^ two days later, at the end of the experiment (*P* < 0.0001, ANOVA-RM), without the applied light regime showing any effects. However, uptake rates remained higher than those measured at 3.8 µM.

### Nitrate reductase activity

**Fig. 3:**
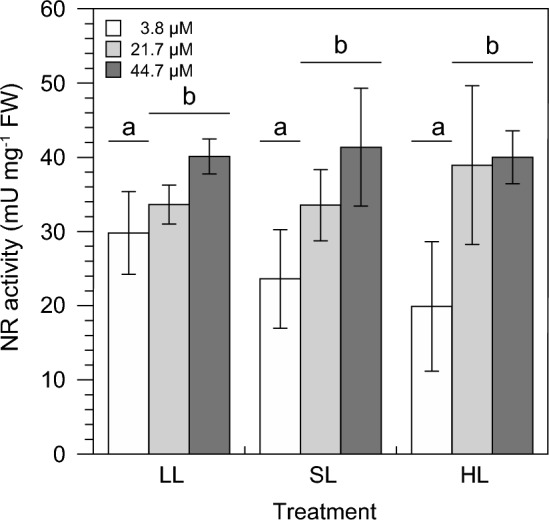
Nitrate reductase (NR) activities of *Ulva rigida* after three days of an exposure to different nitrate concentrations in seawater and growth light intensities. Error bars denote SDs of three replicates per treatment (*n* = 3). Different lower case letters above the columns indicate statistically significant differences of means between treatments (*P* < 0.05, 2-way ANOVA, Tukey HSD). For details of experimental treatments, see Fig. 1 and Table 1

On the third day of the experiment, NR activity was regulated by nitrate concentrations in seawater alone (*P* = 0.0018, 2-way ANOVA) and not by growth light intensities (*P* = 0.1959, 2-way ANOVA) or an interaction (*P* = 0.3602, 2-way ANOVA). Activities of NR were the lowest at 3.8 µM (24.4 ± 7.5 mU mg^−1^ FW) and increased when nitrate concentration was 21.7 µM (35.5 ± 6.6 mU mg^−1^ FW) and 44.7 µM (40.5 ± 4.6 mU mg^−1^ FW) but without showing significant differences between these two nitrate treatments (Fig. 3).

### Photosynthetic performance

**Fig. 4:**
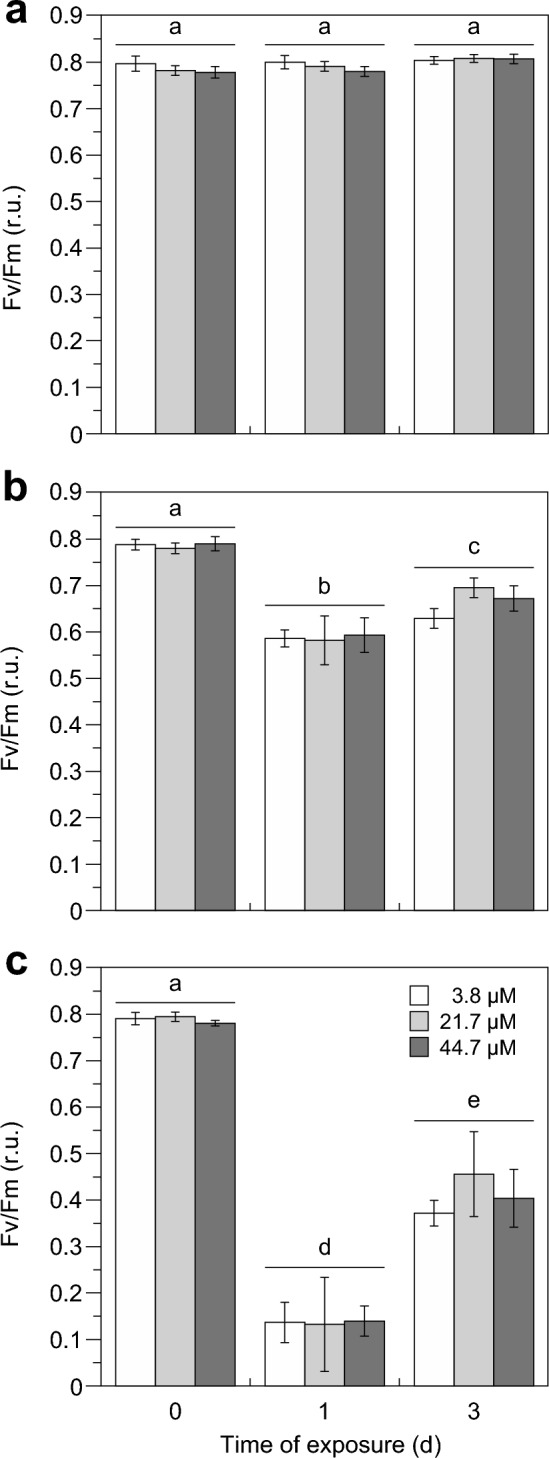
Changes in Fv/Fm of *Ulva rigida* exposed to **A** limiting (LL: 52 µmol photon m^−2^ s^−1^), **B** saturating (SL: 612 µmol photon m^−2^ s^−1^) and **C** high light intensities (HL: 1,111 µmol photon m^−2^ s^−1^) at three seawater nitrate concentrations (3.8, 21.7 and 44.7 µM) over three days. Error bars represent SDs of three replicates per treatment (*n* = 3). Different lower case letters above the columns indicate statistically significant differences of means between treatments (*P* < 0.0001, 2-way ANOVA, Tukey HSD). For details of experimental treatments, see Fig. 1 and Table 1

After the ‘pre-experimental’ acclimation and before *U. rigida* was exposed to different nitrate and light regimes, *U. rigida* showed Fv/Fm values of 0.787 ± 0.012 (*P* = 0.4709, 2-way ANOVA) that remained constant under LL throughout the experiment (*P* = 0.4802, 1-way ANOVA-RM; Fig. 4A). After 8 h under SL on the first day, Fv/Fm decreased by 25% to 0.587 ± 0.033 (*P* < 0.0001, 2-way ANOVA-RM) but increased again by 13% to 0.665 ± 0.035 over the following two days (Fig. 4B). A strong decline in Fv/Fm by 83% to 0.137 ± 0.057 (*P* < 0.0001, 2-way ANOVA-RM) was measured after 8 h in the HL treatment (day 1). However, Fv/Fm markedly increased by 200% to 0.411 ± 0.067 (*P* < 0.0001, 2-way ANOVA-RM) following two days in the HL treatment (Fig. 4C).

**Fig. 5:**
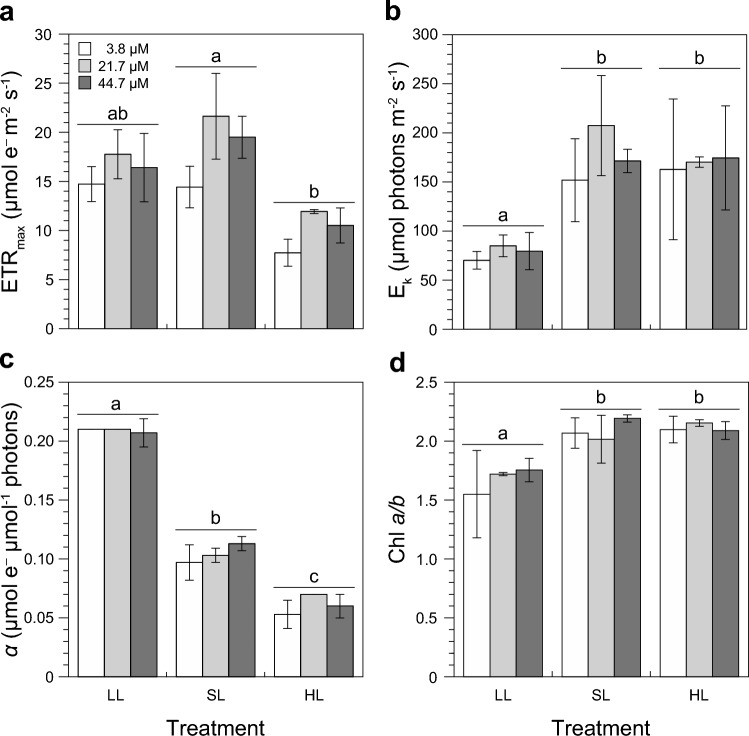
Photosynthetic parameters and chlorophyll a/b ratio of *Ulva rigida* after three days of exposure to three different light conditions and three nitrate concentrations in seawater. **A** Maximum electron (e^–^) transport rates (ETR_max_). **B** Light saturation points (*E*_k_) of electron transport rates. **C** Initial slopes of electron transport rate vs. irradiance (ETR-*E*) curves. **D** Chlorophyll *a/b* ratios. Error bars denote SDs of three replicates per treatment (*n* = 3). Different lower case letters above the columns indicate statistically significant differences of means between treatments (*P* < 0.05, 2-way ANOVA, Tukey HSD)

Photosynthesis by *U. rigida* was solely influenced by the growth light intensities—neither nitrate concentration (all *P* > 0.05, 2-way ANOVA) nor an interaction between the two factors (all *P* > 0.05, 2-way ANOVA) had an effect on photosynthetic parameters. Although ETRmax of LL- (16.3 ± 1.5 µmol e^–^ m^−2^ s^−1^) and SL-grown (18.5 ± 3.7 µmol e^–^ m^−2^ s^−1^) *U. rigida* were similar after three days, ETRmax decreased significantly in the HL treatment (9.4 ± 1.5 µmol e^–^ m^−2^ s^−1^; *P* = 0.0086, 2-way ANOVA; Fig. 5A). The light intensity at which ETRs of *U. rigida* were saturated (*E*_k_) reflects a typical light-dependent pattern: *E*_k_ under LL (78 ± 7 µmol photons m^−2^ s^−1^) was the lowest and increased under both SL (177 ± 28 µmol photons m^−2^ s^−1^) and HL (159 ± 18 µmol photons m^−2^ s^−1^; *P* = 0.029, 2-way ANOVA; Fig. 5B). The electron transport efficiency, which is represented by the initial slope of ETR-*E* curves (α), gradually decreased with an increasing growth light intensity (*P* < 0.0001, 2-way ANOVA; Fig. 5C). LL-grown algae showed highest α values (0.209 ± 0.002 μmol e^–^ μmol^−1^ photons) that were reduced by 50% and 71% under SL (0.104 ± 0.008 μmol e^–^ μmol^−1^ photons) and HL (0.061 ± 0.008 μmol e^–^ μmol^−1^ photons), respectively.

### Chlorophylls

After three days, both Chl *a* and *b* contents of LL-grown algae were higher than those of algae exposed to both SL and HL, which were almost identical amongst each other (both *P* < 0.001, 2-way ANOVA). Neither the content of Chl *a* nor of Chl *b* was affected by seawater nitrate concentrations (both *P* > 0.05, 2-way ANOVA) or an interaction between growth light intensity and seawater nitrate (both *P* > 0.05, 2-way ANOVA). In LL-grown algae, 1.21 ± 0.09 µg Chl *a* mg^−1^ FW and 0.72 ± 0.01 µg Chl *b* mg^−1^ FW were determined. When *U. rigida* was exposed to higher light intensities (i.e. SL and HL), contents of Chl *a* and *b* decreased by 57% (0.69 ± 0.08 µg Chl *a* mg^−1^ FW) and by 46% (0.33 ± 0.03 µg Chl *b* mg^−1^ FW), respectively (data not shown).

The Chl *a*/*b* ratio showed a clear light effect (*P* = 0.0124, 2-way ANOVA) with no influence of the different nitrate concentrations in seawater (*P* > 0.9278, 2-way ANOVA) nor an interaction between both factors (*P* = 0.8154, 2-way ANOVA; Fig. 5D). Chl *a*/*b* ratios in LL-grown algae (1.67 ± 0.11) were lower than in algae grown under both SL (2.09 ± 0.09) and HL (2.11 ± 0.04; Fig. 5D).

### Non-photochemical quenching (NPQ)

**Fig. 6:**
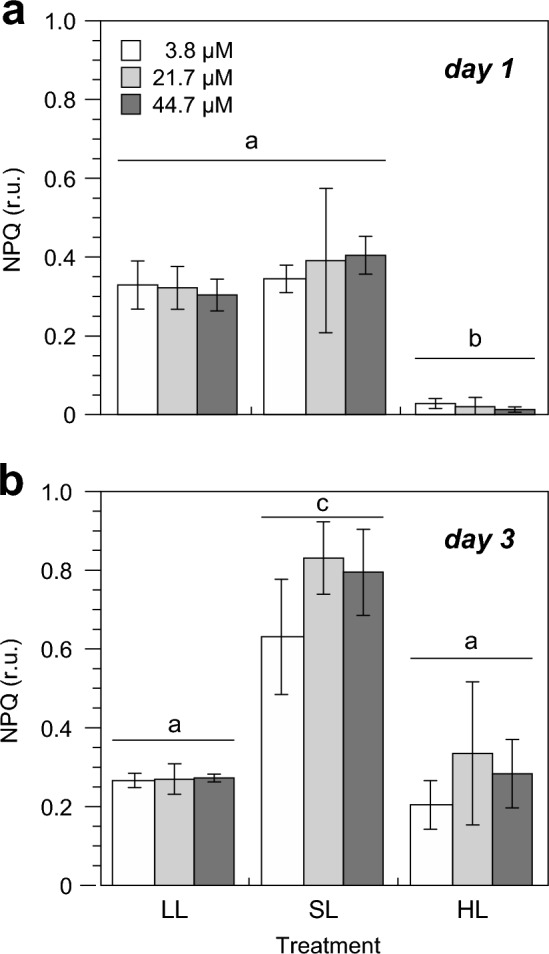
Non-photochemical quenching (NPQ) of *Ulva rigida* exposed to three different light conditions and seawater nitrate concentrations on the first (**A**) and the third day (**B**) of the experiment. Error bars denote SDs of three replicates per treatment (*n* = 3). Different lower case letters above the columns indicate statistically significant differences of means between treatments (*P* < 0.05, 2-way ANOVA, Tukey HSD). For details of experimental treatments, see Fig. 1 and Table 1

NPQ in *U. rigida* was strongly regulated by the growth light intensity alone (*P* < 0.0001, MANOVA) and increased significantly as a consequence of the prolonged light stress on day 3 (*P* = 0.0199, MANOVA). Whilst NPQ remained unchanged compared to LL (0.319 ± 0.047) after eight hours of exposure to SL (0.381 ± 0.100) on day 1, NPQ in the HL treatment declined significantly to 0.021 ± 0.015 (Fig. 6A). Two days later (day 3), NPQ increased significantly to 0.750 ± 0.136 in response to prolonged SL exposure. Although HL also caused a significant increase in NPQ compared to day 1 (0.283 ± 0.117), it did not rise above the level measured of LL-grown algae (0.293 ± 0.025). Neither seawater nitrate concentration (*P* < 0.2559, 2-way ANOVA) nor an interaction between both factors (*P* < 0.4001, 2-way ANOVA) had an effect on NPQ in *U. rigida*.

### Activities of antioxidant enzymes

On the third day of the experiment, both total SOD (35 ± 10 U mg^−1^ protein) and GR (0.17 ± 0.04 U mg^−1^ protein) activities remained unchanged in all light and nitrate treatments compared to LL (SOD: *P* = 0.2632; GR: *P* = 0.6061, both 2-way ANOVA) (data not shown).

## Discussion

The present study reveals that the stimulation of N_i_ assimilation caused by enhanced seawater nitrate concentrations had no effects on photoinhibition and ROS production in *Ulva rigida* to maintain its high growth rates. Thus, photosynthesis and nitrogen assimilation did not affect each other in this important bloom-forming green macroalga, even though both physiological processes are interconnected. Although eutrophication of coastal seawater can have a strong impact on the physiology of marine macroalgae by stimulating photosynthesis and growth (Gordillo 2012), this was not the case for the strain of *U. rigida* used in this study. Its photosynthesis and growth were clearly affected by the incident light intensities, whereas seawater nitrate concentrations solely increased nitrate uptake and reduction, without showing interactions between these two major environmental factors. Therefore, it seems to be unlikely for this *U. rigida* strain that mechanistic interactions between the nitrogen metabolism and photosynthesis based on a common electron share might have an influence on the formation and maintenance of green tides.

### Regulation of the nitrogen metabolism by nitrate reductase activity

Nitrate uptake, internal storage in the vacuoles, its reduction and assimilation are closely coupled processes in green macroalgae. The acclimation of the nitrogen physiology to changing nitrate concentrations in seawater by adjusting the activity of the nitrate-reducing enzyme, NR, strongly controls both uptake and storage by a feedback mechanism (Kennison et al. 2011). At an initial seawater nitrate concentration of 3.8 µM, constant low uptake rates and NR activities (~ 25 mU mg^−1^ FW) indicate that the nitrate taken up from seawater was entirely assimilated rather than internally stored. This implies that *U. rigida* constantly assimilates the available nitrate in the oligotrophic coastal waters of the collection site where nitrate concentrations occasionally exceed 5 µM in winter (Phillips and Hurd 2003; Dean and Hurd 2007; Kregting et al. 2008).

When *U. rigida* was transferred from pre-experimental (3.8 µM) to higher nitrate concentrations (≥ 21.7 µM), the instant increase in nitrate uptake rates (4–6 ×) can be primarily ascribed to the filling of the intracellular nitrate pools (i.e. vacuoles). At this point, the low NR activity resulting from the pre-incubation was not able to reduce nitrate to nitrite as fast as it entered the cells, which results in the storage of nitrate in the vacuoles (McGlathery et al. 1996; Lartigue and Sherman 2005; Kennison et al. 2011). Higher nitrate concentrations increased the NR activities through enhanced transcription of the corresponding gene as demonstrated for *Ulva prolifera* (Guo et al. 2017). Constant uptake rates during the first eight hours after the transfer suggest that nitrate pools were slowly and continuously filled without reaching capacity within this period of time. This is in contrast to other *Ulva* species in which nitrate uptake rates decreased within 3–6 h after having been exposed to higher nitrate concentrations since nitrate pools reached capacity along with maximum NR activities (Lartigue and Sherman 2005; Kennison et al. 2011). Hence, NR activity in *U. rigida* seems to adjust more slowly to increasing nitrate concentrations than other *Ulva* species due to the filling of larger nitrate pools (Naldi and Viaroli 2002; Lartigue and Sherman 2005). Two days later, nitrate pools were filled to capacity as indicated by significantly lower uptake rates and, meanwhile, NR activity increased to the maximum rate of 35–41 mU mg^−1^ FW. This shows that nitrate uptake decreases when NR reached its maximum activity; then uptake rates seem to be equivalent to the maximum NR activity, which, in turn, does not require nitrate to be stored temporarily in the intracellular pools. This indicates that the activity of NR is the rate-limiting step of the entire nitrate-assimilating pathway in *U. rigida*. A ‘negative feedback regulation process’ of uptake, storage and reduction/assimilation was demonstrated to be present in green macroalgae, including *U. rigida* (McGlathery et al. 1996; Naldi and Viaroli 2002; Lartigue and Sherman 2005; Kennison et al. 2011). It seems that the acclimation of *U. rigida* to nitrate concentrations ≥ 21.7 µM is strongly associated with the adjustment of NR activities to control uptake rates.

NR activity is often regulated by the synthesis of the enzyme due to gene expression, which is triggered by different environmental factors (nitrate, light, CO_2_) and the intracellular availability of carbon skeletons (Huppe and Turpin 1994). In *U. rigida*, NR activity was mainly regulated by nitrate, stimulating its synthesis at concentrations ≥ 21.7 µM. Because growth and nitrogen uptake/assimilation were not coupled, *U. rigida* seems to be saturated with N_i_ under oligotrophic conditions. In nitrate-replete *Ulva lactuca*, excess N_i_, which is not used for growth, was allocated to protein synthesis, increasing enzyme contents and other nitrogen-containing metabolites (Teichberg et al. 2007). Because growth rates of *U. rigida* did not seem to be associated with internal N_i_ pools as in other *Ulva* species, *U. rigida* can proliferate and potentially form green tides even under oligotrophic conditions due to constant N_i_ assimilation rather than by N_i_ pool sizes (Teichberg et al. 2010). Therefore, eutrophication does not seem to be the primary factor for its fast growth when other factors (e.g. light) are limiting.

### Photoprotection and photoacclimation are regulated by light to sustain macroalgal growth

NPQ is the most important photoprotective mechanism that dissipates excessively absorbed light energy as heat to protect the photosynthetic apparatus, mainly PSII, from photodamage (Wilhelm and Selmar 2011). In *Ulva rigida*, PSII-photodamage under sudden light stress as measured by the fast decline in Fv/Fm can be ascribed to the inactive NPQ. In both *Ulva fasciata* and *U. lactuca*, a similar sharp decline in Fv/Fm and NPQ was accompanied with a rapid and immense degradation of the PSII-intrinsic D1 protein after these macroalgae was transferred from low to high light (80–1500 µmol photons m^−2^ s^−1^). After 2 h of high light stress, 50–70% of the initially active D1 protein were damaged and degraded before the rapidly declining Fv/Fm levelled off at values ~ 0.1 (Carr and Björk 2007). Since the decrease in Fv/Fm did not entirely reflect the damages of the D1 protein by showing lower values than the actual D1 protein contents, an additional mechanism was apparently active: some of the remaining, non-degraded PSII-RCs were still active and fully functional in photochemistry, whilst others were inactive but not damaged. Photochemically active PSII are thought to be responsible for the low Fv/Fm values measured in *U. lactuca* (0.07) and *U. fasciata* (0.09–0.13), which are similar to 0.137 under HL in *U. rigida* (this study). Inactive, non-damaged PSII-RCs can dissipate excess excitation energy from the ‘still’-coupled LHCII as heat and, in turn, protect neighbouring PSII from photoinactivation to avoid further structural damages (Öquist et al. 1992; Franklin 1994; Franklin and Larkum 1997; Chow et al. 2002). Despite the lack of NPQ, photoprotection by both photoinhibition and PSII-RC quenching seems to be very effective not only in *U. fasciata* and *U. lactuca* but also in *U. rigida*.

The production of superoxide anions (O_2_^*–^) remained low in *U. rigida* due to effective photoprotection. Neither SOD nor GR activities increased after three days of light stress, which indicates that ROS production was low and could be sufficiently detoxified by the Mehler-ascorbate peroxidase pathway. This finding further supports the currently discussed hypothesis that the photodamage of PSII is caused by a light-induced inactivation of the PSII-associated oxygen-evolving complex rather than by ROS produced due to an over-reduction of the primary electron acceptor (i.e. Q_A_) or a charge recombination in PSII (Ohnishi et al. 2005; Murata et al. 2012). The present study suggests that photoinhibition in combination with the PSII-RC quenching seem to be crucial to *U. rigida*’s ability to respond to high light stress when the NPQ processes such as the xanthophyll cycle are ‘inactive’.

After two days of exposure to light stress, the marked photoacclimation of PSII activity for *U. rigida* measured by the recovery of Fv/Fm can be attributed to the fast restoration of the PSII-RCs by the PSII repair cycle that exceeds the rate of photodamage. Since higher rates of photodamage and degradation of the D1 protein are followed by a faster restoration of the PSII-RCs under HL compared to SL, the rates of the PSII repair via de novo synthesis of the D1 protein apparently depend on the amount of damaged and degraded PSII-RCs that lack the D1 protein (Tyystjärvi 2013). The restoration of PSII activity by the PSII repair cycle apparently influences the regain of NPQ: the increase in NPQ under both SL and HL could result from the build-up of the proton gradient (ΔpH) across the thylakoid membrane due to the increasing PSII activity. After the lumen pH dropped below ~ 6, violaxanthin de-epoxidase is activated and converts violaxanthin to eventually zeaxanthin in the xanthophyll cycle. Concurrently, the PSII-associated PsbS and LHCSR proteins in *Ulva* become protonated, which allows the zeaxanthin-PsbS/LCHSR aggregates to dissipate excess excitation energy in LHCII as heat rather than to transfer excitons to other chlorophylls and form singlet oxygen (Mou et al. 2013; Zhang et al. 2013). However, how far this scenario of NPQ reactivation is involved in the photoacclimation under prolonged high light stress in *Ulva* requires further investigation. As a long-term photoacclimation to light stress, *U. rigida* reduced the absorption cross section of PSII (i.e. size of LHCII) after three days of expose to both SL and HL significantly. Smaller PSII antennae, which are indicated by high Chl *a/b* ratios of 2.09–2.11 under SL and HL compared to 1.67 under LL, decrease the absorption of light and limit energy transfer to PSII to protect PSII-RCs from light-induced oxidative stress.

All these short-term photoprotective and long-term photoacclimation mechanisms together allow *U. rigida* to sustain its photosynthetic activity, which has direct consequences for its growth. Whilst photoprotection and -acclimation maintained a high photosynthetic activity under SL to enable growth rates of 20% d^−1^, the HL-induced photoinhibition was clearly responsible for the growth reduction of *U. rigida*’s by 60% (RGR = 12% d^−1^). Because high growth rates of ≥ 20% d^−1^ favour the proliferation, light intensities ranging between 300 and 600 µmol photons m^−2^ s^−1^ seem to be optimal for the formation of green tides by *U. rigida* (Beer et al. 2000; Rautenberger et al. 2015). Although such light intensities were shown to favour the proliferation of *U. prolifera* by relatively high growth rates (20–40% d^−1^), its combination with other local environmental conditions, such as optimal temperature and salinity regimes, allow the green macroalga’s to bloom (Xiao et al. 2016; Wu et al. 2018, 2022). Nevertheless, growth rates of 9–12% d^−1^ may allow *U. rigida* to outcompete most macroalgae, whose growth rate are usually lower (Teichberg et al. 2010).

It is striking that the different, ecologically relevant nitrate concentrations in seawater had no supporting effect on both photoprotection and photoacclimation under SL and HL in *U. rigida*, which suggests that these mechanisms were saturated at seawater nitrate concentrations of 3.8 µM. This is supported by the N_i_-dependent pattern of photoacclimation in *Ulva rotundata*: whilst PSII activity was able to acclimate to high light stress over seven days under sufficient N_i_ supply (15 µM NH_4_^+^), photoinhibition persisted when ammonium concentration (1.5 µM NH_4_^+^) remained low (Henley et al. 1991). As the pattern of high light acclimation in N_i_-replete *U. rotundata* was very similar to that of *U. rigida* in the present study, it can be concluded once again that *U. rigida* was sufficiently N_i_-supplied at nitrate concentrations ≥ 3.8 of nitrate to support the PSII repair cycle with sufficient amino acids for the biosynthesis of the D1 protein (Litchman et al. 2002; Davison et al. 2007).

### Does increased nitrogen assimilation function as a photoprotective mechanism in *Ulva rigida*?

In microalgae, the non-linear relationship between the photosynthetic ETR and oxygen production or carbon incorporation rates at supersaturating light intensities was ascribed to ‘alternative’ sinks of photosynthetic electrons, protecting them from photoinhibition (Wilhelm et al. 2006; Napoléon and Claquin 2012). Whilst photorespiration is largely suppressed in green algae (e.g. *Ulva*) and diatoms that employ an efficient carbon-concentrating mechanism, large amounts of photosynthetic electrons can be transferred to oxygen at the acceptor side of PSII under high light (Claquin et al. 2004; Waring et al. 2010). In nitrate-replete diatoms, excess photosynthetic energy can be channelled to nitrate reduction, resulting in the release of ‘non-nutritionally’ reduced nitrate into the surrounding environment (Lomas and Gilbert 1999).

Whether or not the stimulation of the nitrogen metabolism can function as a photoprotective mechanism by redirecting a higher proportion of photosynthetic electrons to nitrate assimilation is still unclear for macroalgae despite the hint that high seawater nitrate affects the relationship between ETR and oxygen production in *U. rigida* (Cabello-Pasini and Figueroa 2005). Since photosynthesis provides eight electrons by Fd_red_ to NiR and glutamate synthase (GS) to assimilate one mole of nitrate in photosynthetically active tissue, an increase in nitrogen assimilation rates (e.g. due to eutrophication) may require a higher amount of photosynthetic electrons. However, in this study the conspicuous lack of any interaction between the nitrate and light treatments suggests that there is no substantial increase in electron consumption by NiR and GS under higher nitrate concentrations. This is because NR activity, as the rate-limiting step of the entire nitrogen-assimilating pathway, controls the rates of the subsequent reactions steps, including those of NiR and GS. Since NR activity in *U. rigida* increases only slowly and moderately (~ + 50%) to higher nitrate concentrations, NiR and GS activities cannot rise significantly to create a higher electron sink than at 3.8 µM. Moreover, an excretion of nitrate into the surrounding seawater was not detected under any experimental treatment, which suggests that only a basic and constant amount of photosynthetic electrons is delivered to assimilate nitrogen, regardless the incident light intensity. Furthermore, a decreased PSII activity under photoinhibition (at SL and HL) and the remaining low photosynthetic activity under chronic photoinhibition (at HL) photosynthetic electrons are only provided in sufficient quantities to be utilised by the Calvin-Benson cycle. Therefore, nitrate assimilation does not seem to act as a photoprotective mechanism in *U. rigida*.

## Conclusion

*Ulva rigida* is a green macroalgal species that is able to form large green tides in coastal marine areas. This can cause huge ecological and economic problems when green tides persist and spread out (Smetacek and Zingone 2013). Because growth of *U. rigida* is strongly controlled by light intensity rather than the seawater nitrate concentrations resulting from N_i_-saturation at oligotrophic conditions (3.8 µM), effective photoprotection and photoacclimation are required to maintain its photosynthetic activity and thus high growth rates. Apparently, a purposeful damage and degradation of the D1 protein (i.e. photoinhibition) in combination with the PSII-RC quenching reduces the ‘excitation pressure’ in PSII efficiently when heat dissipation of excess light energy by NPQ is inactive. By these short-term photoprotective mechanisms, *U. rigida* was able to respond rapidly to light stress before long-term photoacclimation could become effective. The re-invigoration of heat dissipation by regaining NPQ activity along with the reduction of the PSII absorption cross section allowed *U. rigida* to keep its daily growth rates high. These physiological mechanisms combined can be regarded to as a pre-requisite for the formation of green tides. Being arranged in dense mats, *U. rigida* is exposed to a steep light gradient. Whilst the light-exposed top and ‘sub-canopy’ layers could maintain high growth rates due to effective photoprotection and -acclimation, efficient light capture strategies (e.g. increased PSII antenna sizes) may allow *U. rigida* to reach sufficient growth rates at bottom layer despite light-limitation. The present study demonstrates that the high phenotypic plasticity of *U. rigida* with respect to the regulation of the photosynthetic activity is crucial to maintain its massive growth. Although proliferation can cause enormous and economic losses in coastal marine ecosystems, it could be also beneficially applied to remove or neutralise pollutants under bioremediation.

## Data Availability

The data sets generated and/or analysed during this study are available from the corresponding author on reasonable request.

## Abbreviations

ETR: Electron transport rate
HL: High light
LL: Low light
NiR: Nitrite reductase
NR: Nitrate reductase
SL: Saturation light
SOD: Superoxide dismutase
